# Interventions and platforms that direct lymphangiogenesis to restore physiological homeostasis and enhance immunoregulation

**DOI:** 10.1038/s41536-026-00472-z

**Published:** 2026-05-12

**Authors:** Lauren G. Porter, Mikayla S. Jackson, Robert S. Oakes

**Affiliations:** 1 Department of Biomedical Engineering, University of Delaware, Newark, DE USA; 2 Veterans Affairs Maryland Health Care System, United States Department of Veterans Affairs, Baltimore, MD USA

**Keywords:** Cancer, Immunology

## Abstract

The lymphatic system maintains physiological homeostasis through constant immunosurveillance, immune cell trafficking, and regulation of interstitial fluid flow. Lymphatic dysfunction is associated with a wide range of pathologies, including cancer metastasis and lymphedema. Aberrant lymphatic structure also contributes to chronic wounds and transplanted organ rejection. These functional and structural deficits have inspired strategies for lymphatic vasculature modulation and tissue engineering to regulate immune functions during disease and injury rehabilitation. Lymphangiogenesis—the process of lymphatic vessel growth— is central to the success of these strategies and has broad potential if harnessed for therapeutic intervention and tissue integration. Here we review the opportunities and obstacles for biomolecular pathway modulation, nanotechnology, and tissue engineering to promote or inhibit lymphangiogenesis.

## Introduction

**Fig. 1:**
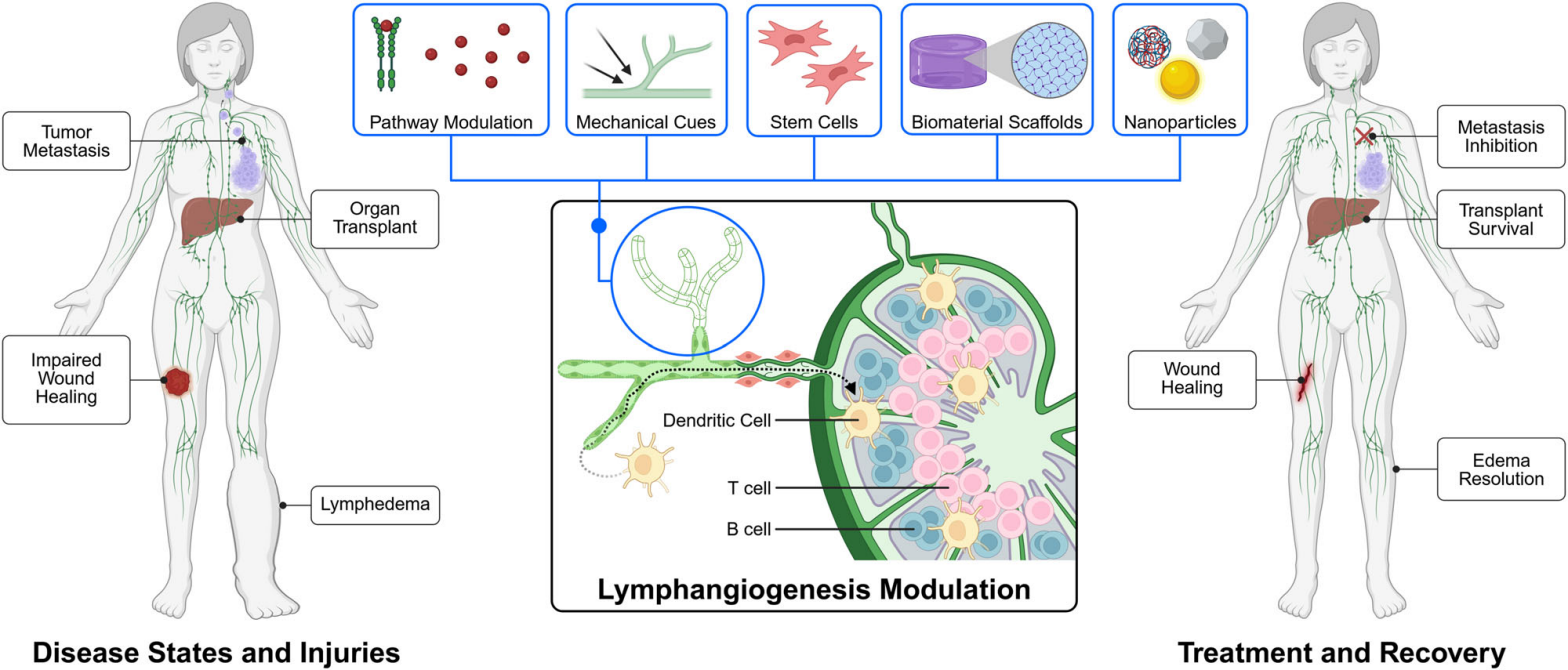
Control of lymphangiogenesis for treating disease and regenerative medicine. The lymphatic system maintains immunological homeostasis through constant immunosurveillance, immune cell trafficking, and regulation of interstitial fluid flow. Lymphatic dysfunction is associated with a wide range of pathologies, including cancer metastasis and lymphedema. Poor lymphatic integration also contributes to impaired wound healing and rejection of transplanted organs^1^. This has inspired strategies for modulation of lymphangiogenesis—the process of lymphatic vessel growth—to improve clinical outcomes for therapeutic intervention and regenerative medicine. These include growth factor engineering and delivery, control of mechanical cues, delivery and differentiation of stem cells, use of biomaterial scaffolds to direct lymphatic vessel growth, and nanoparticles for imaging and targeting lymphatic networks. Through modulation of lymphatic networks, these strategies impact the function of innate and adaptive immune cells within the lymphatic system and the overall balance of systemic immunoregulation. The outcome of this lymphangiogenic modulation is improved treatment and injury recovery.

The lymphatic system is the main immunological organ system present throughout the body^1^. It includes a network of lymphatic vasculature (LV) that collects and transports interstitial fluid and immune cells. Once this milieu enters the LV, this protein-rich fluid known as lymph is carried in a unidirectional manner through regional lymph nodes (LNs) where immune cells reside at a high density^2^. This fluid clearance complements the nutrient supply from blood vasculature throughout most tissues, however LV remained understudied until a series of seminal discoveries in the 1990s^3–7^. Emerging research has shown that the growth and remodeling of LV—known as lymphangiogenesis—plays a vital role in prevalent pathologies (e.g., cancer) and injury rehabilitation^8^. This review highlights novel findings on lymphatic vessels, with emphases on immune regulation and harnessing lymphangiogenesis via therapeutic and tissue engineering platforms (Fig. 1).

### Structure of lymphatic vasculature

**Fig. 2:**
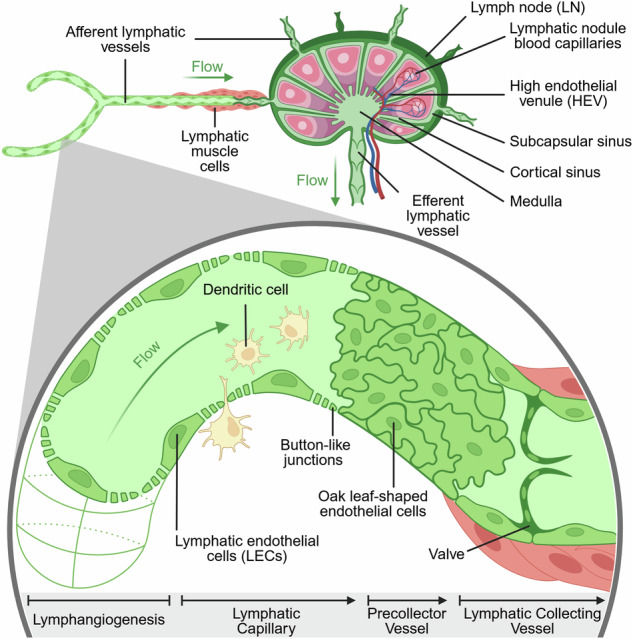
Lymphatic vasculature networks and LECs facilitate immune cell and signal transport. The LV collects and transports fluid, cells, and macromolecules in a unidirectional manner through regional LNs. Draining interstitial fluid is denoted as lymph once it enters LV. Lymphatic capillaries are highly permeable vessels characterized by discontinuous, button-like junctions between individual LECs and anchoring filaments connecting them to the surrounding ECM^11,12^. Capillaries flow into pre-collector vessels, which have a discontinuous basal membrane and are characterized by the occasional presence of valves. Pre-collector vessels flow into collector vessels, which possess a complete basal membrane and a layer of LMCs surrounding them. Collector vessels have continuous zipper-like junctions between LECs and are characterized by regular valves throughout, which prevent the backflow of lymph and divide the collector vessels into a series of chambers known as lymphangions^233^. The lymphatic vessels directing flow towards LNs are known as the afferent vasculature, while lymphatic vessels directing flow away from LNs are known as the efferent vasculature.

The LV is present throughout most vascularized tissues in the body and is primarily composed of lymphatic endothelial cells (LECs). Specialized lymphatic vessels exist in specific organs, including the intestine and cardiac tissue^9,10^. Though the LV has similarities to the blood vascular system, it is characterized by unique physiological structures (Fig. 2) and cellular markers that distinguish it from blood vessels. Understanding the anatomical structure and cellular markers of the LV allows for better therapeutic modulation of its responses.

#### Lymphatic anatomy

The anatomical structure of the lymphatic system allows for the efficient transport of lymph throughout the body. Like the blood vascular network, the LV is organized in a hierarchical structure, flowing from lymphatic capillaries to larger precollector and even larger collecting vessels. Lymphatic capillaries are blind-ended, highly permeable vessels characterized by discontinuous, button-like junctions between individual LECs, allowing for fluid, cells, and molecules to enter from the surrounding interstitial space^11^. When interstitial fluid pressure increases, the surrounding extracellular matrix (ECM) stretches, pulling on anchoring filaments connected to LECs and opening the intercellular junctions^12^. Lymphatic precollector vessels are surrounded by a discontinuous basal membrane and are characterized by the occasional presence of valves. LECs in pre-collector vessels are more tightly joined together than in initial vessels, but still are permeable to specific cell types, including CCR10 + T cells in inflammatory states^13^. In contrast, lymphatic collecting vessels have a complete basal membrane and a layer of lymphatic muscle cells (LMCs) surrounding them. They have continuous zipper-like junctions between LECs, and typically do not absorb fluid. Collecting vessels are also characterized by regular valves throughout, preventing the backflow of lymph and dividing them into chambers known as lymphangions. The opening and closing of valves are dependent upon pressure gradients and intrinsic LMC contraction, which has been found to be modulated via histamine and nitric oxide (NO) production. In addition, surrounding skeletal muscle contraction and arterial pulsations contribute to lymphatic vascular flow^14,15^. The largest collecting vessel in the body is the thoracic duct, which flows into the subclavian vein via the lymphovenous valve^16^. Overall, despite the functional and structural differences between the blood and lymphatic vascular networks, they are intimately connected.

#### Lymphatic cell identification

The identification of unique LEC markers in the late 1990s began a new era of study of LV since it could be easily distinguished from blood vasculature for the first time. First, vascular endothelial growth factor receptor 3 (VEGFR-3), originally called fms-like tyrosine kinase 4 (FLT4), was identified by the Alitalo group in 1995 as a molecular signature for lymphatic vessels^3^. VEGFR-3, encoded by the associated gene *VEGFR3*, is a dimeric receptor found primarily on the surface of LECs, but can be expressed by other cell types in certain contexts^17^. Its activation triggers intracellular tyrosine kinase phosphorylation, initiating further signaling cascades promoting LEC differentiation and migration in embryonic development and lymphangiogenesis in mature tissue^18,19^. In addition, lymphatic vessel endothelial receptor 1 (LYVE-1) was identified in 1999 as a CD44 homolog on the surface of lymphatic vessels. LYVE-1 is an integral membrane glycoprotein that binds to both soluble and immobilized hyaluronan with high selectivity^6^. When immune cells generate a hyaluronan glycocalyx, LYVE-1 mediates their migration towards, entry into, and intraluminal crawling within lymphatic capillaries^20^. Furthermore, the membrane mucoprotein podoplanin (PDPN) was identified as an additional lymphatic marker by the Alitalo group in 1999^21^. PDPN is expressed by a variety of cell types, but notably is absent from blood endothelial cells, making it a valuable target for LEC identification. Finally, the transcription factor prospero homeobox protein (PROX1) was identified in 1999 as an additional lymphatic signature, as the expression of its associated gene, *Prox1*, is critical for the differentiation of venous endothelial cells into LEC progenitors in embryonic development^7,22^. More recently, Srinivasan et al. identified a feedback loop between PROX1 and VEGFR-3 in LEC progenitor cells^23^. Specifically, VEGFR-3 activation regulates the expression of *Prox1*, which is critical for the maintenance of LEC identity. By uncovering lymphatic cellular markers, researchers not only increased their understanding of LV development but also created methods for the identification and isolation of LECs for the first time. These discoveries empowered and created a field of study focused on the lymphatic system.

### Mechanisms of lymphangiogenesis

In healthy individuals, after embryonic development, most lymphatic vessels are quiescent and do not undergo lymphangiogenesis, with the exception of in the intestine, in the ovaries, and during wound healing or inflammatory responses^8,24,25^. There is emerging evidence that lymphangiogenesis occurs in the endometrium during phases of the menstrual cycle, but this remains unclear^26^. Mechanisms of lymphangiogenesis have been reviewed extensively, but broadly, it is governed by several signaling cascades, metabolic pathways, and physical and mechanical cues. Understanding key regulators of lymphangiogenesis informs the development of pro- and anti-lymphangiogenic therapies for the treatment of disease.

#### Lymphangiogenic signaling axes

Vascular endothelial growth factor C (VEGF-C) was first identified in 1996 by the Alitalo group as the predominant activating protein of VEGFR-3^4^. VEGF-C is widely considered to be the primary signaling molecule driving lymphangiogenesis. This vital growth factor is a dimeric, secreted protein linked by disulfide bonds that binds to VEGFR-3 and induces its autophosphorylation. Extracellular proteolytic processing of VEGF-C produces a mature, homodimeric, non-disulfide-linked form that is also capable of binding to VEGFR-2 and VEGFR-2/VEGFR-3 heterodimers^27^. VEGF-C is crucial for lymphatic growth, proliferation, and migration, as knockdown of *Vegfc* is embryonically lethal in mice and results in the complete absence of LV formation^28^. VEGF-D is also capable of binding to VEGFR-2 and VEGFR-3, but it is not essential for the development of normal LV^29^. In addition to members of the VEGFR family, VEGF-C and VEGF-D are able to bind to neuropilin-2 (NRP2), a receptor protein expressed in veins and LV. NRP2 was found to be required for the formation of lymphatic capillaries in a murine model^30,31^. In addition to members of the VEGF family, additional signaling axes have been found to promote lymphangiogenesis. First, chemokine receptor CXCR4 binding to its associated ligand, CXCL12, was found to promote LEC migration and growth in vitro and in vivo^32^. Moreover, angiopoietin-1 and 2 (ANG1, ANG2) and their corresponding receptor tyrosine kinases (TIE1, TIE2) have also been found to regulate lymphangiogenic growth. TIE2 is expressed in LECs and is stimulated by both ANG1 and ANG2, inducing lymphatic proliferation^33–35^. The mechanism of TIE1 involvement in lymphangiogenesis is less defined, but reduction of TIE1 expression induced abnormal lymphatic development in mouse embryos, particularly jugular lymphatic sac dilation^36,37^. In addition, several bioactive lipids have been identified as lymphangiogenic regulators, including sphingosine 1 phosphate (S1P). S1P was found to initiate LEC migration and tube formation, as well as stimulate ANG2 secretion from LECs^38–40^. Additional growth factors, chemokines, and molecules have been found to modulate lymphangiogenesis, which have been reviewed extensively^41,42^.

#### Cellular metabolism

Recent studies have shown that metabolic pathways are additional crucial regulators of lymphangiogenesis, primarily by impacting the epigenetic downregulation of *Prox1* and thus the differentiation of LEC progenitors. First, fatty acid β oxidation (FAO) was found to promote lymphangiogenesis by producing acetyl-CoA, which is then utilized by the histone acetyltransferase p300 to increase the accessibility and selectivity of *Prox1*, promoting its expression^43^. PROX1 was shown to autoregulate its own transcriptional activity by increasing LEC expression of the enzyme CPT1A, which enhances FAO and acetyl-CoA production. Another critical component of cellular metabolism involved in lymphangiogenesis is lipid droplet degradation via lipophagy. Impaired lipophagy decreased mitochondrial productivity and FAO, decreasing acetyl-CoA levels and preventing *Prox1* expression^44^. Furthermore, mitochondrial respiration was determined to be an additional regulator of LV development. In a murine model, inhibition of mitochondrial complex III in vitro and in vivo initiated epigenetic downregulation of *Vegfr3* and *Prox1*, preventing LEC differentiation^45^. In addition, the oxidation of ketone bodies was also identified as a pro-lymphangiogenic stimulus. Ketone body oxidation (KBO) was found to sustain the tricarboxylic acid cycle via acetyl-CoA production, enabling nucleotide synthesis during LEC replication^46^. Finally, mammalian target of rapamycin complex 1 (mTORC1) was recently established as a crucial regulator of cellular metabolism in LECs, as well as impacting *Prox1* expression. mTORC1 was found to control expression of *Myc*, which impacts expression of hexokinase 2 and glutaminase, key enzymes in glycolysis and glutaminolysis, respectively. As a result, inhibition of mTORC1 caused decreased lymphatic capillary growth and collecting vessel differentiation in a murine model^47^. Overall, elucidating metabolic regulators will enable lymphangiogenic modulation and open new avenues for lymphatic engineering approaches.

#### Physical and mechanical cues

LECs are responsive to mechanical cues, and studies have elucidated several mechanical forces as important regulators of lymphatic growth and function. First, increased interstitial fluid pressure was found to induce lymphangiogenesis by elongating LECs and increasing VEGFR-3 phosphorylation^48^. Second, as lymph enters lymphatic capillaries by following pressure gradients, it generates transmural flow. This flow has been found to stimulate LECs to increase CCL21 secretion and upregulate the expression of intercellular adhesion molecule 1 (ICAM-1) and E-selectin, which in turn increases dendritic cell (DC) transmigration towards lymphatic vessels^49^. Third, LECs exposed to fluid shear stress due to lymph flow experience increased VEGFR-3 activation, and high VEGFR-3 levels increased LEC sensitivity to shear^50,51^. In addition, fluid shear stress and cyclic strain were found to impact LEC alignment and morphology in vitro^52^. Finally, fluid and oscillatory shear stresses have been found to be crucial for lymphatic valve formation^53,54^. It is vital for in vitro studies of the LV to incorporate these physical and mechanical cues in their observations to model physiological conditions accurately. Understanding the roles of interstitial fluid pressure, transmural flow, shear fluid stress, cyclic strain, and oscillatory shear stress on lymphangiogenesis and lymphatic pumping offers additional insights into the regulation of the LV.

### Lymphatic vasculature in immunological homeostasis

LV has three primary functions: regulating immune responses, absorbing dietary lipids, and maintaining fluid homeostasis^9,24,55^. The interactions between the LV and immune cells are constantly impacting innate and adaptive immune responses.

#### Innate immune regulation

LV has a fundamental role in the regulation of innate immunological responses. Innate immune cells and signals are actively transported toward the LV along chemotactic gradients^56^. Upon interaction with antigen, DCs upregulate CCR7 expression, which attracts them towards CCL21+ LECs. In addition, the expression of CXCL12 by LV has been shown to attract CXCR4+ DCs and neutrophils^57–59^. Innate immune cells enter lymphatic capillaries via button-like junctions. One mechanism for this is mediated by the interaction of LYVE-1, expressed on the surface of LECs, with the hyaluronan glycocalyx of DCs and macrophages^60^. Once inside the lymphatic capillary, innate immune cells perform intraluminal crawling since lymph flow velocity is insufficient at this stage for effective cell transport. As they reach collecting vessels, lymphatic pumping generates increased lymphatic flow and the innate cells are then passively transported towards LNs^61–63^. In addition, S1P has been shown to regulate innate immune cell migration via LV^64–66^. Under homeostatic conditions, innate cell migration occurs without integrin binding, however, innate immune cell binding integrins (e.g., macrophage-1 antigen, Mac-1) are upregulated by LECs during inflammation^11,57,59,67–70^. It must be noted that while other types of innate immune cell transport via LV are well established, there are unknowns regarding the regulation of active macrophage travel through LV versus clearance via local macrophage apoptosis^71^.

#### Adaptive immune regulation

Antigen-presenting cells (APCs) interact with lymphocytes to initiate an adaptive immune response^72^. LV and LECs regulate this innate-adaptive interaction through immunomodulatory pathways like Clever-1^73^. Like innate cells, T cell migration is also regulated through CCL21/CCR7 and S1P/S1PR1 signaling^74–80^. These pathways direct T lymphocytes to enter the LN via high endothelial venules (HEVs) or afferent lymphatic vessels, whereupon they meet APCs. However, there is emerging evidence that APCs may interact with circulating T cells within the lymphatic capillaries, prior to reaching the LN^81^. Naïve T cells exit the LN via efferent lymphatic vasculature following a S1P1 gradient, continuing through the lymphatic circulation and eventually entering the blood circulation again via the thoracic duct^78,82^. If antigen presentation occurs, T cells upregulate CD69, which reduces S1PR1 expression. This prevents LN egress until differentiation, whereupon S1PR1 is upregulated and they can then exit, returning to the site of pathogen to execute effector function^83^. The mechanisms governing B cell trafficking via LV have some pathway overlap with T cells, but are less defined^84^. Naïve B cells rely on CXCR4/CXCL12 signaling, while antigen-activated B cells upregulate CCR7^85^. Interestingly, B cell egress via efferent lymphatic vessels is independent of S1P signaling^86^.

#### LEC antigen presentation

In addition to regulating the trafficking of other immune cells, recent studies have elucidated intrinsic LEC immune functions^87^. Namely, in murine models, LECs can act as APCs through the expression of both major histocompatibility complex (MHC) class I and class II molecules^88–90^. Moreover, LN stromal cells have been shown to present antigen to T cells and induce tolerance in murine models^91,92^. Furthermore, LECs within the LN have been found to capture antigen and “archive” it over a time period of several weeks for subsequent delivery to APCs for CD8 + T cell activation^93^. In a murine model of melanoma, LECs were found to cross-present antigen to tumor-specific CD8 + T cells, driving their deletion and promoting tumor growth^94^. In addition, it was shown that LEC presentation of MHCII may prevent autoimmunity in aging mice by increasing regulatory T cell (Treg) proliferation^95^. Further studies are needed to translate these findings on LEC and lymphatic stromal cell antigen presentation capacity to human biology and in the context of disease. However, this fascinating evidence suggests that lymphatic vessels both direct and facilitate systemic immune responses.

### Lymphangiogenesis in disease

Dysfunction of the lymphatic system and lymphangiogenesis are associated with a myriad of pathologies, including neurodegenerative diseases, cardiovascular diseases, colorectal diseases, cancer, lymphedema, transplant rejection, and impaired wound healing^1,8^. For example, cancer progression exploits excessive lymphangiogenesis, lymphedema lacks sufficient lymphatics, and wound healing requires balanced lymphangiogenesis^96,97^.

#### Cancer

**Fig. 3:**
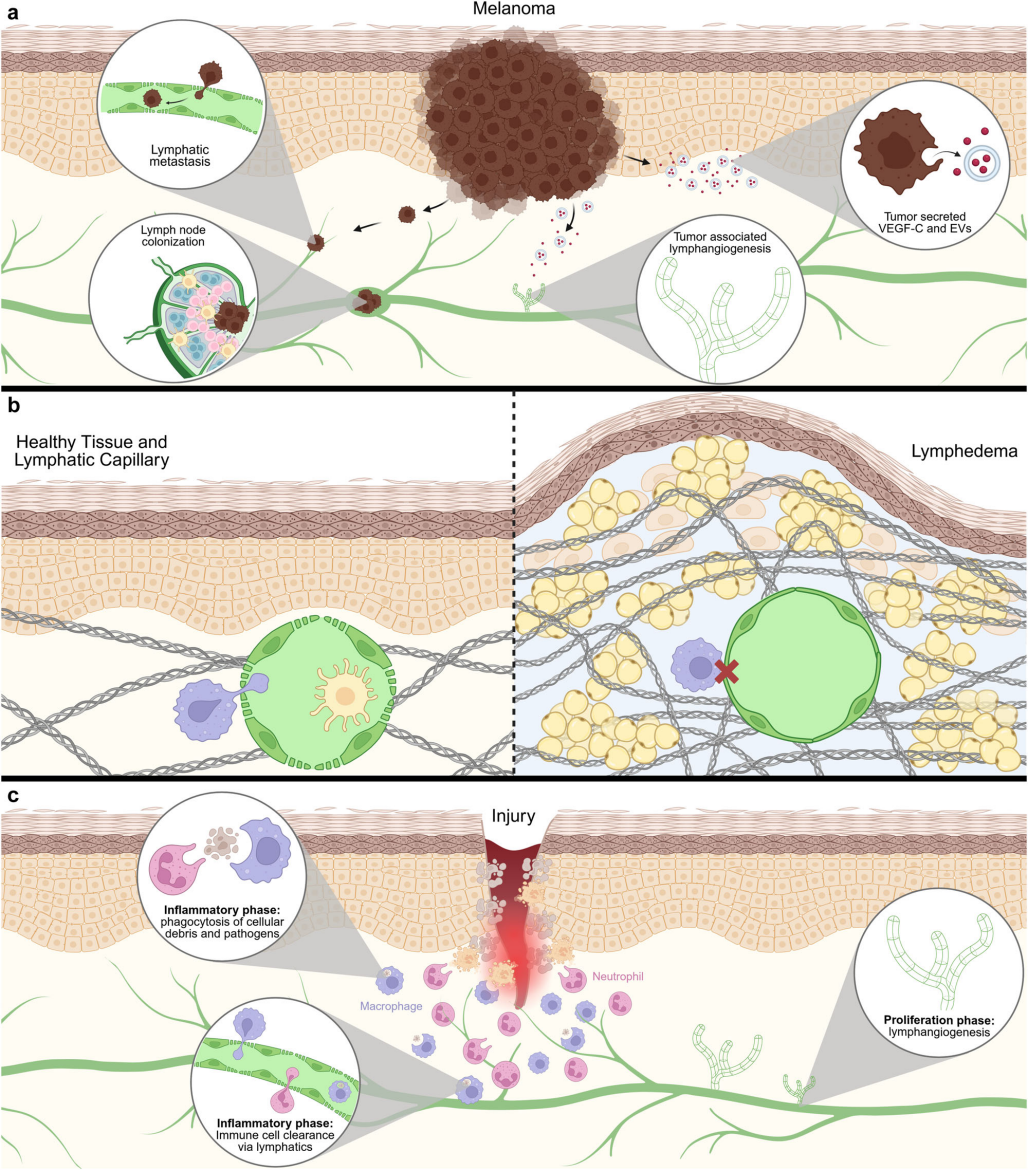
Disease prognosis and injury resolution are dependent on lymphatic function. **a** In cancer, lymphatic vessels provide a route for metastasis from the primary tumor site to distal tissues, which is often observed in the closest draining LN (also known as the sentinel lymph node)^103^. There are several known chemokine gradients that mediate active transport of cancer cells into lymphatic capillaries, including CCL21/CCR7 and CCL21/CXCR3 signaling^110,112,113^. Tumor cells secrete soluble factors, including VEGF-C, that induce tumor-associated lymphangiogenesis, which provides increased pathways for metastatic tumor cells to travel throughout the body and colonize additional tissue. Many studies have shown a positive correlation between LVD and distant metastases in a variety of cancer types. Increased VEGF-C has been correlated with an immunosuppressive environment in LECs by inhibiting T cell migration and activation^88^. **b** In lymphedema, dysfunctional lymphatic drainage leads to the accumulation of fluid, adipose tissue, and fibrotic tissue, leading to localized swelling and inflammation. Lymphedematous tissue is characterized by increased expression of DAMPs that activate TLRs on innate immune cells. The DAMP-TLR cascade induces a chronic pro-inflammatory response via the production of TGF-β and nitric oxide by macrophages^124,125^. However, the lack or dysfunction of LV in affected body parts reduces the transport of activated innate immune cells to LNs, inhibiting adaptive immune responses and creating an immunosuppressive environment via the accumulation of regulatory T cells, further promoting adipocyte accumulation^127^. **c** In the inflammatory phase of wound healing, immune cells accumulate at the site of the wound and phagocytose cellular debris and pathogens. Lymphatic vessels provide a route for the clearance of these immune cells away from the site of the wound^130^. In the proliferation phase, novel lymphatic networks are formed via the process of lymphangiogenesis. The two dominant hypotheses on lymphangiogenesis in wound healing are the “sprouting” hypothesis—LECs sprout from filopodia on existing networks at the site of injury—and the “self-organization” hypothesis —individual LECs migrate into the site of injury, guided by interstitial fluid flow, and organize into vascular networks^132^. It is likely that both theories contribute to lymphangiogenic growth in different phases of the wound healing process.

Tumors induce lymphangiogenesis, which enhances tumor cell metastasis (Fig. 3a)^98–101^. Tumor-associated (TA) lymphangiogenesis is implicated in many cancer types and the quantification of lymphatic vascular density (LVD) is a prognostic factor in cancer staging^102^. TA lymphatics are irregular, enlarged, and facilitate the transport of tumor cells and tumor-secreted exosomes and soluble factors^8^. Mechanisms of TA lymphangiogenesis have been previously reviewed^103,104^. In brief, tumor cells and tumor-associated macrophages (TAMs) secrete VEGF-C, which induces LEC growth and proliferation at the primary tumor site as well as in distal locations^98,105^. Recent findings suggest that sentinel lymph node (SLN) tumor colonization induces pro-tumor immune tolerance that promotes further metastasis^100,106,107^. This colonization is driven by LEC cytokine secretions —an adopted mechanism from healthy homeostatic immunity^108–111^. Specifically, LEC production of CCL21, driven by tumor necrosis factor α (TNFα), has been shown to increase CCR7+ tumor cell metastasis and proliferation^112^. Increased CXCR3 expression by tumor cells has been found to correlate with increased inflammation and lymphatic metastasis, associated with LEC expression of CXCL11 and CCL21^110,113^. Upregulation of programmed death ligand 1 (PD-L1) on LECs supports Tregs and an immunosuppressive, pro-tumor microenvironment^88,114,115^. Finally, TA lymphatic vessels have been found to upregulate CXCL12, recruiting CXCR4 + , CD8 + T cells. The egress of tumor specific T cells from the tumor limits their ability to execute effector function against cancer cells, limiting the effects of immunotherapies^116^. Thus, while lymphatics are necessary for anti-tumor immune responses, lymphangiogenesis is generally associated with cancer progression and worsening prognoses^94,102,103,117–119^.

#### Lymphedema

Lymphedema is a condition defined by impaired lymphatic drainage, causing the accumulation of fluid and adipose tissue, leading to localized swelling and inflammation. (Fig. 3b). Lymphedema can be categorized as primary (i.e., genetic origin) or secondary (i.e., acquired) where damage from trauma, surgery, or radiation therapy alters or destroys lymphatics^120^. Furthermore, emerging data suggest that obesity can induce secondary lymphedema^121^. This lack of transport leads to impaired immune function, increased susceptibility to infection, and dysfunctional wound healing^1^. The immunological impacts of lymphedema have been previously reviewed^122,123^. In brief, lymphedematous tissue is characterized by increased expression of damage associated molecular patterns (DAMPs) that activate toll-like receptors (TLRs) on innate immune cells^124^. This induces a chronic inflammatory phenotype in macrophages at the site of edema^125,126^. Paradoxically, the lack of LV in affected tissues reduces the transport of activated innate immune cells to LNs, thus also inhibiting adaptive immune responses and creating an immunosuppressive environment^127,128^.

#### Wound healing and transplant integration

The complex biological process of wound healing is triggered in any condition or medical procedure where an injury occurs. Wound healing consists of three phases: (i) inflammation, (ii) proliferation, and (iii) remodeling (Fig. 3c)^129^. In the inflammation phase, immune cells accumulate at the site of the wound and secrete growth factors that promote lymphangiogenesis^130^. In proliferation, granulation tissue is formed with integrated blood and lymphatic vessels^129,131^. Hypotheses on existing lymphangiogenic “sprouting” and de novo lymphatic “self-organization” exist for superficial and deep tissue wound healing^132–135^. It is likely that both theories contribute to lymphangiogenic growth in different phases, but additional data is needed. Chronic wounds share several immunological features with lymphedema, thus it is postulated that impaired lymphatic drainage limits wound healing^136^. Chronic challenges also impact organ transplantation where lymphangiogenesis can enable tolerogenic immune responses that promote graft survival, but may also compromise a graft if growth is excessive^137,138^. This dichotomy, along with those listed above, supports the need for therapeutics and platforms that manage lymphangiogenesis alongside immune responses.

## Lymphangiogenesis as a therapeutic intervention

**Fig. 4:**
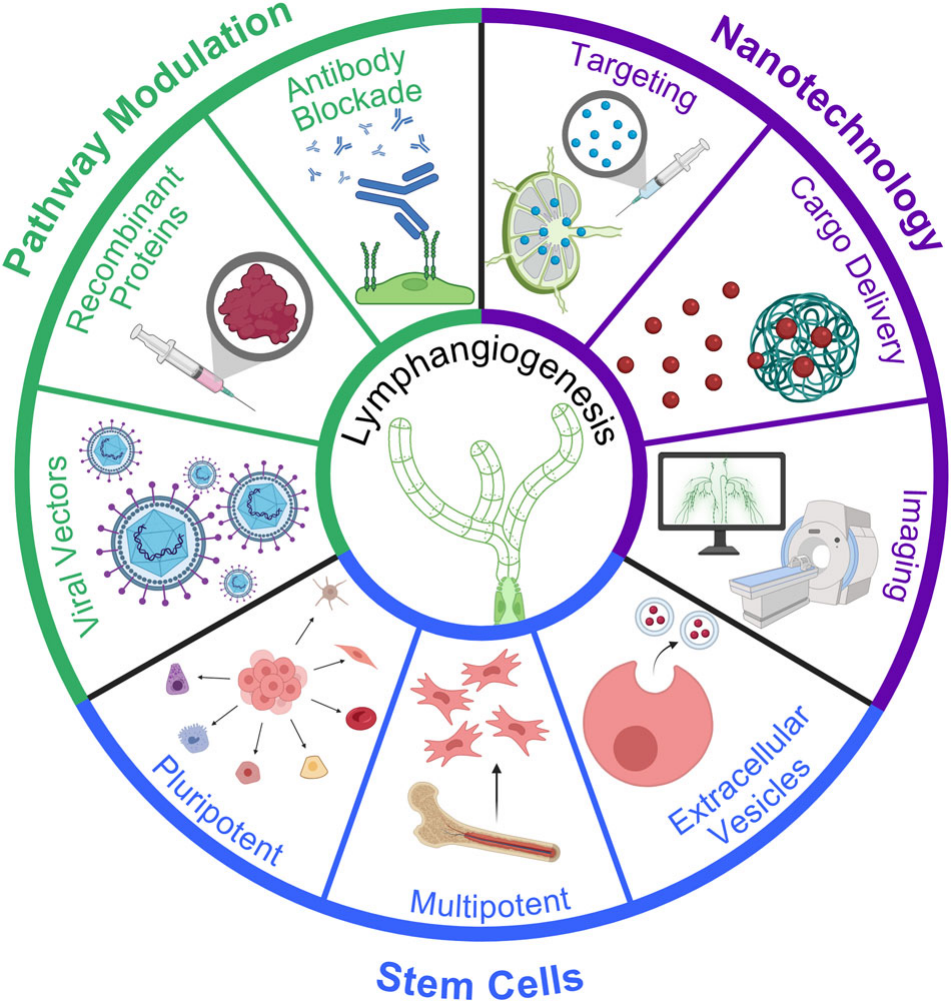
Regulation of lymphangiogenesis through pathway modulation, nanotechnology, and stem cells. Establishing therapeutic interventions to enhance or inhibit lymphangiogenesis and lymphatic remodeling could impact clinical outcomes for numerous pathologies. Modulation of lymphangiogenic signaling pathways has been accomplished through lentiviral and adenoviral vector administration, delivery of recombinant proteins, and antibody blockade^144,147,152^. Nanotechnology has been explored for targeted delivery to lymph nodes, controlled lymphangiogenic and therapeutic cargo release, and imaging of lymphatic vessels and LV networks^159^. Stem cells with pluripotent and multipotent capacities from autologous, allogeneic, and xenogeneic sources have been explored for promoting lymphangiogenesis^184^. This category is subdivided into cell therapies and cell-derived therapies (e.g., extracellular vesicles). Many of these approaches are designed to complement therapeutic (e.g., cancer immunotherapy) or regenerative medicine (e.g., organ transplantation) strategies. Harnessing lymphangiogenesis will require an integrated approach that leverages multiple tools within this set, as well as future technologies.

As outlined above, lymphatic dysfunction and lack of lymphangiogenic control contribute to poor clinical outcomes in multiple contexts. Thus, modulation of lymphatic growth through lymphangiogenic pathway regulation, nanoparticle (NPs) enhanced targeting and imaging, and stem cell transplantation are promising avenues to enable novel and improve existing clinical interventions (Fig. 4).

### Lymphangiogenic pathway modulation

There are multiple interwoven signaling cascades that regulate lymphangiogenesis, as described in section “Lymphangiogenic signaling axes”. These biomolecular pathways can be harnessed to promote or inhibit lymphatic growth using a variety of established methods, including the use of viral vectors to alter gene expression, recombinant protein administration, and antibody blockade.

#### Viral vectors

Viral vectors are a widely established method of gene transfer, used in pre-clinical and clinical treatments for cancer, cardiac diseases, and gynecological disorders^139–141^. The Alitalo lab performed a series of seminal studies using adenoviral vectors (AVVs) to modulate the VEGF-C pathway to upregulate lymphangiogenesis^142,143^. A recent AVV study sequestered VEGF-C signaling in mice, which decreased tertiary lymphoid organ formation, *Ccl21a* expression, and improved transplant survival^138^. In a murine lymphedema model, Michalaki et al. used a lentiviral vector encoding VEGF-C expression to increase lymphatic vessel contraction^144^. Jiang et al. utilized adenoviral ANG1 administration in a murine model of lymphedema, which ameliorated lymphatic drainage and decreased tail swelling and cutaneous thickness in mice lacking hypoxia-induced factor 2α (HIF2α)^145^. Overall, viral vectors have been harnessed to both increase and impede local lymphangiogenesis via modulation of signaling pathways.

#### Recombinant protein administration

The localized injection of recombinant protein isoforms has shown promise for modulating lymphangiogenesis in vitro. VEGF-C156S, a form of VEGF-C with selective VEGFR-3 binding, was first synthesized in 1998^146^. This mutant protein was utilized by Cui et al. to induce local lymphangiogenesis in a murine model of orthotopic lung transplantation^147^. The authors found that induction of therapeutic lymphangiogenesis decreased allograft rejection and contributed to increased wound healing. However, one study found the use of exogenous VEGF-C in a murine model of lymphedema did not significantly improve tissue edema^148^. While this approach has been used in limited therapeutic scenarios, there is broad potential of recombinant protein administration in other pro-lymphangiogenic contexts.

#### Antibody blockade

Antibody blockade utilizes antibody binding to stop signaling cascades^149^. Huggenberger et al. utilized VEGFR-3 blockade to prevent lymphangiogenesis in a model of chronic skin inflammation, which increased inflammatory cell accumulation^150^. A study in an orthotopic human cervical cancer xenograft model found that VEGFR-3 antibody blockade decreased TA lymphangiogenesis as well as metastasis in mice^151^. In addition, Kampen et al. treated acute myeloid leukemia (AML) patient samples with VEGF-C antibodies in vitro and found that blockade suppressed AML colony formation and induced anti-tumor T cells^152^. In a murine model of lymphedema, Kim et al. found that decreased S1P concentration contributed to disease progression. The authors utilized anti-P selectin treatment, resulting in decreased tail swelling and reduced inflammation^153^. Furthermore, a phase 1 clinical trial aimed at evaluating the safety of VEGF-C antibody blockade for cancer treatment was initiated in 2014^154^. Conference proceedings indicate that the treatment was well tolerated, but full data have yet to be published^155^. Overall, antibody blockade of the lymphangiogenic signaling pathways offers a prospective treatment for cancer, which could inform treatments for other diseases.

### Nanotechnology approaches

Another strategy for therapeutic lymphangiogenesis is the use of nanotechnology approaches. Nanotechnology has been widely utilized for the improved detection and treatment of lymphatic metastasis in cancer by leveraging the unique properties of NPs to accumulate in LV and LNs^156^. While cancer pathology was well-aligned with pioneering nanotechnology approaches, other lymphatic-associated pathologies will benefit from these advances.

#### Targeting the lymphatic system

Tuning NP size, charge, and other properties allows them to specifically target the LV and LNs. Studies on NP size have found that 10–80 nm in diameter is beneficial for passive transport to lymph nodes^157–159^. Rao et al. found that anionic particles with a negative surface charge were most successful in lymphatic accumulation^160^. A variety of materials have been utilized for NP fabrication for lymphatic targeting, ranging from metals to biological molecules —such as proteins and micelles—to synthetic polymers like poly-lactic glycolic acid (PLGA)^161–163^. In addition, Gracia et al. exploited the conjugation of adjuvant to cholesterol NPs to enhance lymphatic uptake^164^. Kapnick et al. recently presented an alternative approach by directly injecting diffusion-limited particle depots into LNs to coordinate adaptive immune responses, which was well-tolerated in non-human primates^165^. Thus, customizing the attributes of NPs allows for highly localized delivery to the LV and LNs.

#### Cargo delivery

As an extension of their ability to specifically target the lymphatic system, NPs have been uniquely harnessed to deliver cargo directly to the LNs and LV, including contrast agents for imaging, pharmaceutical drugs, and biological molecules^166^. For example, surface-functionalized NPs have been used to target specific cell subtypes within the lymphatic system^167,168^. In a study focused on transcriptional modulation, Zhang et al. utilized lipid mRNA-laden NPs encoding VEGF-C for lymphangiogenic treatment of myocardial infarction^169^. In cancer, NP delivery of photosensitizers for photothermal therapy has been used for localized ablation, with NPs utilizing lymphatics to target LN metastases. Photosensitizers including verteporfin, indocyanine green lactosome, and gold have been effectively packaged in NPs for lymphatic targeting^170–172^. In chronic wound healing, Xie et al. used cerium-containing bioactive glass NPs to enhance LEC migration, proliferation, and tube formation^173^. Overall, NP-mediated cargo delivery is a powerful approach that exploits the permeability and transportive properties of lymphatics.

#### Imaging techniques

The LV is notoriously difficult to image via traditional techniques. The targeted delivery of imaging contrast agents via nanotechnology has been found to enhance and improve a myriad of existing imaging modalities. Ultrasound (US) imaging of the lymphatic system was enhanced by Kato et al., who developed a novel delivery technique using nanobubbles to target specific LNs. In addition to imaging, the study used fluorescent dye as a model cargo for delivery, and enhanced delivery to specific LNs was achieved^174^. Niu et al. utilized hybrid PLGA-iron oxide NPs to enhance US imaging of metastatic LNs^166^. In addition, several different fluorescently tagged NPs have been utilized to image the LV^175–177^. Xu et al. completed joint computed tomography/fluorescent imaging of lymphatic vessels in a murine model utilizing lipid NPs as contrast agents^178^. Another approach used NPs for photoacoustic imaging of LNs, which could also be leveraged to visualize the LV^179^. Finally, magnetic resonance imaging of DC trafficking through the LV was enhanced with gold NPs^180^. While NPs are widely used to improve imaging of LNs, sensitive and high-resolution imaging modalities for lymphatic vessels remain in development.

### Stem cell approaches

Stem cells are progenitors that differentiate into multiple cell populations under defined conditions^181^. Stem cells have been investigated for their potential to both induce lymphangiogenesis in existing LEC networks and for their ability to differentiate into LECs and develop into lymphatic vessels de novo^182,183^. In a landmark study in 2009, Conrad et al. first characterized the differentiation of multipotent mesenchymal stem cells (MSCs) into LECs in vitro when exposed to VEGF-C, identifying lymphatic markers including LYVE-1, PDPN, PROX1, and VEGFR-3^184^. Additional studies have investigated pluripotent and multipotent stem cells as a therapeutic tool to enhance lymphangiogenesis for the treatment of diseases.

#### Pluripotent stem cells

Induced pluripotent stem cells (iPSCs) have been investigated in limited contexts for lymphangiogenic treatments. Several studies have differentiated LECs from iPSCs in vitro, which led to formation of lymphatic capillary networks^182,185^. In one study, Lee et al. generated LECs from iPSCs and then implanted the cells into wounds on mice. The authors observed accelerated wound healing in mice treated with iPSC-derived-LECs over wild type LECs^186^. These findings indicate the potential for iPSCs as a therapeutic for wound healing, but lymphangiogenic potential requires future studies.

#### Multipotent stem cells

Multipotent MSCs are able to differentiate into LECs and have been used in both preclinical and clinical studies for the treatment of lymphedema. The most common MSC sources are adipose derived mesenchymal stem cells (AdMSCs) and bone marrow derived mesenchymal stem cells (BMMSCs). In animal models of lymphedema, treatment with MSCs of human, allogeneic, and autologous sources have been shown to restore lymphatic drainage, decrease tissue swelling, and increase LVD^136,187–191^. Across three clinical trials, treatment with autologous BMMSC transplantation improved lymphedema symptoms, including reducing edema volume and decreasing patient pain^192–194^. Interestingly, across two clinical trials, treatment with autologous AdMSC transplantation was found to decrease patient pain but did not significantly reduce the volume of edema^195,196^. While promising, additional studies with larger patient cohorts at later stages of lymphedema are needed to assess treatment durability. In wound healing, MSCs are a therapeutic strategy used in pre-clinical models of diabetic wounds, transplants, and surgical injuries, showing that MSCs stimulate lymphangiogenesis^136,197–199^. These emerging findings highlight the potential for MSCs to promote wound healing.

#### Stem cell-secreted extracellular vesicles

In addition to direct treatment with MSCs, extracellular vesicles (EVs) from MSCs cultured under specific conditions have been utilized as a therapeutic intervention. MSC EVs have been found to increase LEC viability, proliferation, migration, and tube formation via the delivery of microRNAs. Multiple studies have shown that exposing LECs to EVs secreted by MSCs treated with VEGF-C in vitro increases their lymphangiogenic properties, including lymphatic sprouting^200,201^. Yang et al. found that LECs treated with EVs from MSCs cultured in hypoxic conditions had increased lymphangiogenic properties in vitro^202^. These studies show that stem cell EVs are an additional route for the modulation of VEGF-C signaling that can be harnessed for therapeutic potential. These cell-derived signals, along with the cellular and recombinant proteins listed above, indicate feasibility for tissue engineering LV networks.

## Tissue engineering approaches to modulate lymphangiogenesis

**Fig. 5:**
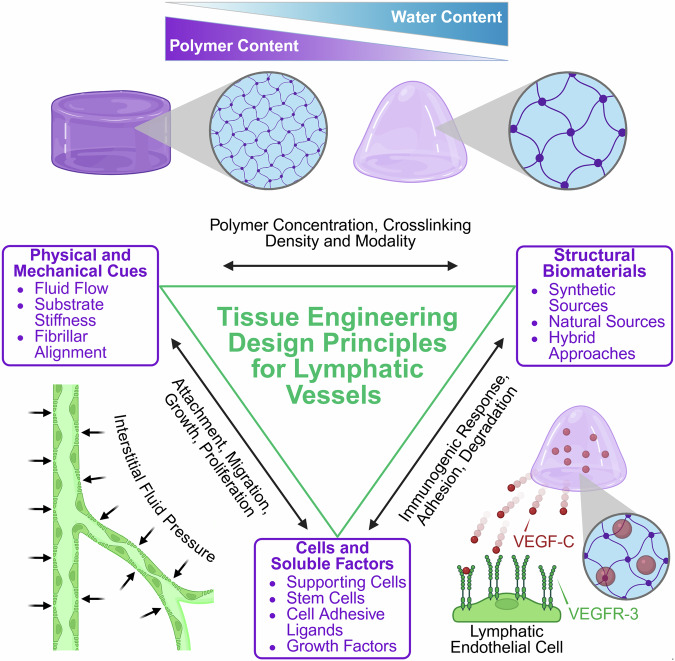
Tissue engineering design principles for lymphatic vessels. Tissue engineering constructs are capable of modulating lymphangiogenesis in vivo and creating physiologically relevant in vitro models for preliminary screening^216^. Tuning physical, mechanical, biological, and chemical properties of biomaterials allow for the customization of lymphatic growth and remodeling in tissue engineering approaches. Customization of biomaterials via material selection, polymer concentration, crosslinking density, and crosslinking mechanism allows for control of physical and mechanical properties, including substrate stiffness^210^. This also provides the necessary structural support for lymphatic vessel growth. These cues interact with cargo encapsulated within the biomaterials, including cells and soluble factors, to direct cell behavior, including growth and migration. The cells and soluble factors interact with the biomaterial components to direct degradation, initiate immune responses, and to enhance cellular adhesion to artificial scaffolds. Collectively, these parameters are interconnected in their regulation of tissue engineering for lymphatic vessels.

Tissue engineering, or the combination of biological and synthetic materials to replace or enhance native tissues in the body, can be utilized as a strategy for the modulation of lymphangiogenesis. The implantation of artificial tissue constructs to enhance and direct lymphangiogenesis have been explored, and there are multiple factors that can be tuned to tailor the location and degree of lymphatic growth. Tissue engineering approaches can customize lymphangiogenesis through physical and mechanical cues, biomaterials, cells, and soluble factors (Fig. 5). In addition, tissue engineering constructs can be utilized to create in vitro models that better recapitulate the lymphatic physiological environment for preliminary research.

### Physical and mechanical cues

Cells receive mechanical stimuli that direct their behavior, from growth and proliferation, to migration and cell death. The process of recognizing mechanical information and translating it into biological signals is known as mechanotransduction. As discussed previously, the lymphatic system is subject to a variety of important physical and mechanical cues that direct its behavior and development.

#### Fluid flow

The lymphatic system is inherently responsive to mechanical forces, including shear stress and pressure gradients^203^. Researchers have mimicked these physiological cues to enhance lymphatic sprouting in vitro. In a key study on LV formation, Helm et al. found that exposure to fluid flow significantly enhanced LEC formation into complex vascular networks in 3D cell culture^204^. Planas-Paz et al. further elucidated LEC phenotypic control when they determined that interstitial fluid pressure upregulates LEC proliferation^48^. In a co-culture model with MSCs, cyclic stretching mimicked pressure gradients to induce LEC alignment, creating a fully lumenized vessel^205^. These studies illustrate the importance of shear, pressure, and direction for lymphangiogenesis, thus informing tissue engineering strategies for ex vivo conditioning prior to implantation and for in vitro disease modeling.

#### Substrate stiffness

In development, LEC progenitors migrate from areas of high stiffness to areas of low stiffness^206^. As a result, researchers have modulated the mechanical stiffness of biomaterials to optimize an environment for lymphangiogenesis via tuning various parameters, including: crosslinker ratios within a hydrogel, polymer concentration and molecular weight, degradation mechanism (e.g., matrix metalloproteinases) and rate of a material, and control of spatial crosslinking using light-reactive moieties^207–210^. Across these many avenues for customizing substrate stiffness, researchers have found that a soft material with low polymeric density allows for the most robust LV formation because LECs are able to migrate through and remodel their environment^204,211^.

#### Fibrillar alignment

In the development of novel lymphatic networks, the alignment of LECs along a physical guide has been shown to impact cell directionality and growth. The use of electrospun fibers as a scaffold for directing LEC attachment has been investigated in LV engineering. Bouta et al. used synthetic fibers to enhance and organize LEC adhesion, finding that cell migration was optimal in a low-density fiber substrate^212^. Recently, Hadamitzky et al. utilized the commercially available BioBridge collagen fibrillar scaffold to guide lymphatic regeneration in a porcine model of secondary lymphedema. The authors found that the fibers provided a directional substrate for LECs to migrate along^213^. These studies show that providing a physical cue of a fibrillar structure can improve LEC migration and organization, allowing for directed lymphatic growth into desired locations.

### Biomaterials platforms

To tune the previously outlined physical and mechanical properties, a variety of polymeric materials have been utilized as scaffolds for LEC growth. While tissue engineering platforms for lymphangiogenesis without a biomaterial component do exist, biomaterial scaffolds of natural and synthetic origins are widely employed in tissue engineering strategies to enhance cellular adhesion and integration with the host tissue^190,214,215^. In addition to physical properties, the biocompatibility of materials must be considered, including their degradability and immunogenicity.

#### Synthetic polymers

Synthetic polymers are artificially created and utilized for numerous biomedical applications. Advantages of synthetic biomaterials are that they are highly customizable and stable long term in vivo. However, they do not possess any inherent cell adhesive ligands and can cause inflammatory immune responses —this could stimulate lymphangiogenesis in theory, but evidence is lacking^216^. When using fibrillar scaffolds, synthetic polymers also provide enhanced tensile strength^212^. Polyethylene glycol (PEG) is a widely used material because it is immunologically inert and is highly customizable via distinct functionalizations. Hooks et al. utilized PEG to create a hydrogel for in vitro lymphangiogenesis. Using a donor vessel, the PEG scaffolds improved lymphedema progression in a rat model^208^. Overall, synthetic polymers offer a biomaterial platform that can withstand a range of stress and strain in addition to providing tailored crosslinking and functionalization capabilities.

#### Natural polymers

Naturally derived components of the ECM have been widely utilized as scaffolds for tissue engineering because of their intrinsic biocompatibility and biodegradability, as well as their native cell adhesive ligands. However, they can be less customizable than synthetic materials and often degrade quickly in vivo, which can limit tissue integration^216^. Fibrin, an ECM protein, has been widely utilized for lymphangiogenic tissue engineering, as well as in conjunction with another ECM protein, collagen^204,217,218^. In addition to hydrogels, collagen has been utilized as nanofibers and sheets to provide topographical features for LEC adhesion and migration^205,213^. Altogether, natural polymers provide desirable scaffold traits for lymphangiogenic tissue engineering through their biocompatibility and integration with host tissue.

#### Hybrid approaches

Some naturally occurring biomaterials can be functionalized with synthetic molecules to achieve enhanced physical and biochemical properties. One common example in lymphatic tissue engineering is hyaluronic acid (HA), a glycosaminoglycan found in the ECM that binds to the LEC marker LYVE-1 in vivo. It should be noted that HA binding to LYVE-1 is highly dependent on both HA conformation and receptor surface density^60^. Because of its carboxyl and hydroxyl groups, HA can be modified to form ester, amide, and ether bonds, which makes it an ideal candidate for lymphatic tissue engineering^209,211^. In addition to HA, gelatin, a derivative of collagen, has been modified for enhanced crosslinking using glutaraldehyde and methacrylate for lymphatic tissue engineering applications^207,219^. Hybrid biomaterials are advantageous for modulating lymphangiogenesis by harnessing the strength and tunability of synthetic polymers in concert with the biocompatibility and native ligands of natural biomaterials.

### Cells and soluble factors

The incorporation of relevant cell types in tissue engineering constructs mimics the physiological functions of the native organ or tissue of interest. In lymphatic tissue engineering, LECs are widely used, but the inclusion of stem cells, supporting cell types, cell adhesive ligands, and growth factors, either in addition to or instead of LECs, have been shown to enhance lymphatic growth and sprouting both in implanted constructs in animals and in vitro models.

#### Stem cells

As discussed previously, stem cells have been widely utilized in lymphangiogenic interventions because of their differentiation abilities, including into LECs^182^. In lymphatic tissue engineering, stem cells from a variety of sources have been utilized for their lymphangiogenic potential, including AdMSCs, BMMSCs, and dental pulp derived stem cells^190,205^. Because they can be autologously sourced from various tissues, stem cells provide a patient-specific, inherently biocompatible cell population that is desirable for tissue engineering.

#### Supporting cell types

To provide structural support and in situ secretion of growth factors, researchers have included a variety of secondary cell types to enhance artificial lymphatic growth. Fibroblasts have been used as a supporting cell type because of their ability to produce lymphangiogenic factors as well as their ability to secrete ECM proteins^214,218^. In addition, researchers have engineered lymphatic and blood vascular networks in concert by seeding LECs with blood endothelial cells (BECs). In co-culture in vitro models, LECs and BECs organized into distinct vascular structures with unique properties^204,218,220^. Including supporting or feeder cell types in tissue engineering constructs enhances the growth and proliferation of lymphatic vessels by mimicking their native in vivo environment.

#### Cell adhesive ligands

For synthetic biomaterials, the incorporation of cell-adhesion molecules can increase cellular attachment to an artificial scaffold. One of the most commonly used adhesion motifs is arginyl-glycyl-aspartic acid (RGD), an amino acid sequence associated with glycoproteins found in the ECM that enables cellular adhesion via integrin-mediated binding^221^. By including RGD in biomaterials, researchers have observed increased cell integration with scaffolds and improved lymphangiogenic sprouting from LECs^208,209^. This is advantageous for enhancing artificial polymers to better simulate the physiological cues of natural biomaterials.

#### Growth factors

As discussed previously, growth factors such as VEGF-C can enhance lymphangiogenesis. There are multiple modalities for including growth factors within biomaterials, from soaking a scaffold in a solution prior to implantation to developing a novel form of the growth factor that binds to a material (namely, fibrin) that is released over time as the scaffold is degraded^213,217^. The inclusion of growth factors in tissue engineering constructs provides a therapeutic stimulus entwined into the implantable scaffold, which can enhance lymphangiogenesis and incorporation into the surrounding tissue environment. The successful integration of VEGF-C with tissue engineering principles detailed above indicates a valuable scientific and clinical opportunity for improving tissue construct integration.

## Future outlook

As outlined in the prior sections, control of lymphangiogenesis is feasible through pathway modulation, nanotechnology, and stem cell approaches. In addition, tuning physical, chemical, and biological properties allows for the customization of lymphatic tissue engineering in animal and in vitro models. However, there is a lack of lymphangiogenic platforms in clinical care. This translational gap exists for biological and technological reasons. First, data and knowledge of lymphatic system functionality is still in its infancy. With the identification of LEC-specific markers only in the past thirty years, the processes regulating the LV are still being identified. As the role of cellular metabolism in lymphangiogenesis continues to be elucidated, exploiting these metabolic pathways provide additional targets for therapeutic lymphatic modulation. For instance, Garcia-Caballero et al. found that a ketogenic diet was shown to decrease tail swelling, dermal thickening, and LV dilation in a murine model of lymphedema, which poses the question of how diet modifications can be utilized in the treatment of lymphatic dysfunction^46^. In addition, while novel pathways involved in lymphangiogenic signaling continue to be investigated, the translation of this knowledge from the lab to the clinic continues to face challenges. For example, Tian et al. found that inhibition of leukotriene B4 with the drug ubenimex was found to improve lymphedema in mice, resulting in increased lymphatic drainage and reduced swelling^222^. While animal studies showed promise, an ubenimex clinical trial for lymphedema was initiated in 2018 but ultimately deemed unsuccessful due to inconclusive results^223^. One reason for the significant translational gaps is the differences between existing animal models and human physiology. For instance, humans have hundreds of LNs while mice only have approximately twenty-two^224^. Another example is that lymphedema in murine models is often studied in the tail because of the lack of adipose tissue accumulation in mouse limbs, which limits translation to humans^225^. Third, while lymphatic tissue engineering has explored a variety of implantation sites in animals, including the skin, mammary fat pad, limbs, and tails, systematic comparison of lymphangiogenesis in different organs is lacking. Fourth, there is limited data on the role of lymphangiogenesis in regulating the foreign body response (FBR)^226,227^. This is a knowledge gap with high potential given the extensive data on localized immunomodulation of the FBR and the ability to aggregate disease-specific cell types in cancer, autoimmunity, and tissue transplantation^228–232^. Biomaterial scaffolds act as synthetic immune niche in these contexts. Finally, due to the complexities of individual immune systems, lymphangiogenic treatments for cancer, lymphedema, and wound healing do not have a “one-size-fits-all” solution. The development of personalized medicine using genomic and proteomic analyses will likely improve the targeted growth and pruning of lymphatics to match the biology of an individual and their disease status or injury.

## Data Availability

No datasets were generated or analysed during the current study.
